# Evaluation of Poly(vinyl alcohol)–Xanthan Gum Hydrogels Loaded with Neomycin Sulfate as Systems for Drug Delivery

**DOI:** 10.3390/gels9080655

**Published:** 2023-08-14

**Authors:** Diana Serbezeanu, Manuela Maria Iftime, Gabriela-Liliana Ailiesei, Alina-Mirela Ipate, Alexandra Bargan, Tǎchiţǎ Vlad-Bubulac, Cristina Mihaela Rîmbu

**Affiliations:** 1 “Petru Poni” Institute of Macromolecular Chemistry, 41A Grigore Ghica Voda Alley, 700487 Iasi, Romania; ciobanum@icmpp.ro (M.M.I.); gdarvaru@icmpp.ro (G.-L.A.); ipate.alina@icmpp.ro (A.-M.I.); anistor@icmpp.ro (A.B.); tvladb@icmpp.ro (T.V.-B.); 2 Department of Public Health, “Ion Ionescu de la Brad” Iasi University of Life Sciences, 8 Sadoveanu Alley, 707027 Iasi, Romania; crimbu@yahoo.com

**Keywords:** PVA/XG hydrogels, neomycin, mechanical properties, drug delivery systems, antimicrobial activity

## Abstract

In recent years, multidrug-resistant bacteria have developed the ability to resist multiple antibiotics, limiting the available options for effective treatment. Raising awareness and providing education on the appropriate use of antibiotics, as well as improving infection control measures in healthcare facilities, are crucial steps to address the healthcare crisis. Further, innovative approaches must be adopted to develop novel drug delivery systems using polymeric matrices as carriers and support to efficiently combat such multidrug-resistant bacteria and thus promote wound healing. In this context, the current work describes the use of two biocompatible and non-toxic polymers, poly(vinyl alcohol) (PVA) and xanthan gum (XG), to achieve hydrogel networks through cross-linking by oxalic acid following the freezing/thawing procedure. PVA/XG-80/20 hydrogels were loaded with different quantities of neomycin sulfate to create promising low-class topical antibacterial formulations with enhanced antimicrobial effects. The inclusion of neomycin sulfate in the hydrogels is intended to impart them with powerful antimicrobial properties, thereby facilitating the development of exceptionally efficient topical antibacterial formulations. Thus, incorporating higher quantities of neomycin sulfate in the PVA/XG-80/20-2 and PVA/XG-80/20-3 formulations yielded promising cycling characteristics. These formulations exhibited outstanding removal efficiency, exceeding 80% even after five cycles, indicating remarkable and consistent adsorption performance with repeated use. Furthermore, both PVA/XG-80/20-2 and PVA/XG-80/20-3 formulations outperformed the drug-free sample, PVA/XG-80/20, demonstrating a significant enhancement in maximum compressive stress.

## 1. Introduction

Antimicrobial drug delivery systems have garnered considerable interest and recognition in the realm of medicine and pharmaceutical sciences. These innovative systems are designed to deliver drugs effectively while simultaneously combating microbial growth, making them highly sought-after in the field [1,2,3]. The development of such drug delivery systems is driven by the need to address the growing challenge of antimicrobial resistance and improve the therapeutic outcomes of antimicrobial treatments [4]. One of the key advantages of drug delivery systems with antimicrobial properties is their ability to achieve localized and targeted drug delivery [5]. By specifically targeting the site of infection, these systems can minimize systemic exposure and reduce the risk of systemic toxicity. Moreover, localized delivery can also enhance the concentration of the antimicrobial agent at the infection site, thereby improving its effectiveness [6]. This can be particularly beneficial in chronic or persistent infections, where continuous exposure to the antimicrobial agent is required to eliminate the pathogenic microorganisms. By encapsulating or conjugating the antimicrobial agent, its physicochemical properties can be optimized, leading to improved drug delivery and therapeutic outcomes. Overall, drug delivery systems with antimicrobial properties hold immense potential in the field of infectious disease treatment. 

PVA, a synthetic water-soluble polymer, is known for its excellent biocompatibility, non-toxicity, and film-forming capacity [7,8]. It has been extensively studied for various applications due to its versatility and ability to modify its physical properties [9,10,11]. Xanthan gum (XG), on the other hand, is a natural polysaccharide derived from monosaccharides (glucose) via fermentation in the presence of *Xanthomonas campestris* bacteria [12]. It possesses unique rheological and viscoelastic properties, making it an ideal candidate for hydrogel formulations [13], three-dimensional networks formed by hydrophilic polymers with the ability to absorb and retain substantial quantities of water or biological fluids [14,15,16]. PVA (polyvinyl alcohol)/xanthan gum (PVA/XG) hydrogels are a type of biocompatible material that has gained significant attention in various fields, including biomedicine and drug delivery systems [17,18,19,20,21]. Thus, the combination of PVA and XG in hydrogel formulations offers several advantages [13,21,22]. Firstly, it improves the mechanical strength and stability of the hydrogel matrix [20]. The presence of XG enhances the gelation process and provides structural integrity, preventing the collapse or disintegration of the hydrogel [17]. Furthermore, the incorporation of XG can alter the swelling characteristics and effectively regulate the release of encapsulated drugs or bioactive molecules from the hydrogel [23]. The incorporation of oxalic acid, a dicarboxylic acid with distinctive chemical characteristics, plays a significant role in strengthening the PVA/XG hydrogels, resulting in improved mechanical properties and expanding their potential applications [19]. In its presence, cross-linking bonds within the PVA/XG hydrogel network may occur, thus increasing its structural integrity, strength, and durability. The incorporation of oxalic acid into PVA/XG hydrogels also offers the advantage of modulating the hydrogel’s swelling behavior and hydrolytic stability. The interaction between oxalic acid and the polymer chains can influence the degree of swelling and control the release of encapsulated substances. This ability to finely tune the swelling and release characteristics makes PVA/XG hydrogels reinforced with oxalic acid suitable for various controlled drug delivery applications. 

Neomycin, a highly water-soluble antibiotic, is utilized for treating certain bacterial infections [24,25]. It is employed in combination with other antibiotics for superficial ophthalmic infections caused by susceptible bacteria. Additionally, it is used in the curative and prophylactic treatment of otitis externa and bacterial infections affecting skin lesions [26,27]. It may also be used orally or in combination with other antibiotics to treat certain gastrointestinal infections. Pharmacokinetic studies have revealed that the active compound exhibits a relatively short plasma half-life of 3–4 h [28]. However, it is rapidly absorbed in the intestinal tract, suggesting its suitability for utilization in controlled-release formulations. The inclusion of neomycin in drug delivery systems presents a promising strategy for enhancing the treatment of bacterial infections [27,29,30,31]. These systems offer controlled release mechanisms, targeted delivery capabilities, and improved stability specifically designed for neomycin [32]. As a result, they effectively enhance the antimicrobial efficacy of neomycin and contribute to improved patient outcomes. By providing controlled release, the systems ensure a sustained and regulated release of neomycin, optimizing its therapeutic effects over an extended period. Additionally, the targeted delivery feature enables precise delivery of neomycin to the infection site, maximizing its concentration and reducing systemic exposure [27,31]. Furthermore, the improved stability of neomycin within these systems prevents degradation and preserves its potency, ensuring consistent and reliable performance. To date, other studies presented the development of a novel hydrogel dressing (HD) loaded with neomycin sulfate [33]. The authors prepared several neomycin sulfate-loaded HDs by varying the amounts of polyvinyl alcohol (PVA), polyvinyl pyrrolidone (PVP), and sodium alginate (SA) using the freeze–thawing technique. They showed that the neomycin sulfate-loaded HD displayed superior wound-healing effects compared to a commercial product. It effectively facilitated the disappearance of granulation tissue and restored the wound tissue to a normal state. Other authors presented a novel approach to developing neomycin-loaded systems using PVA/pullulan (PULL)/protein/peptide hydrogels, with bovine serum albumin (BSA) or lysozyme as proteins and a tripeptide, reduced glutathione (GSH) as the peptide component [34]. The study aimed to explore the release behavior of neomycin sulfate under simulated physiological conditions. The specific objective of this research was to create porous hybrid hydrogels and investigate their potential for delivering neomycin in a controlled manner. These hybrid hydrogels are well-suited for wound-dressing applications and have the potential to effectively deliver antibiotics [34].

**Scheme 1:**
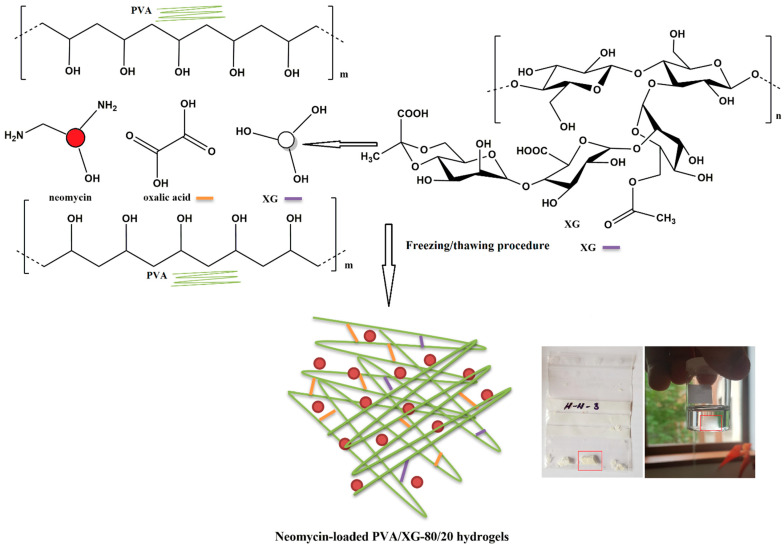
Schematic representation of hydrogel synthesis and drug-loading of hydrogels and images of hydrogel in a dry state and in a hydrated state.

In the present study, neomycin sulfate was loaded into the PVA/XG-80/20 hydrogels as an active compound to treat bacterial infections. In this respect, PVA/XG-80/20 hydrogel was used as a polymer matrix for different amounts of neomycin sulfate. PVA-XG hydrogels cross-linked with oxalic acid were prepared using PVA and xanthan gum as co-polymer networks using the freezing–thawing method followed by thermal treatment, as presented previously in our paper [19]. A schematic illustration of the neomycin-loaded PVA/XG-80/20 hydrogels is given in Scheme 1.

The PVA-XG-80/20 hydrogels and PVA/XG-80/20 hydrogels loaded with different amounts of neomycin were investigated by FTIR, SEM, mechanical testing, swelling behavior, drug release, and antimicrobial activity.

## 2. Results and Discussion

### 2.1. Fabrication of the Hydrogels and Their Structural Characterization

The freezing–thawing method was applied to produce a binary PVA/XG-80/20 hydrogel. Then, the PVA/XG-80/20 hydrogel was successfully infused with the active ingredient (neomycin sulfate) at a temperature of 37 °C in a pH 7.4 phosphate buffer solution, followed by evaporating the excess solvent and drying the sample through lyophilization. The full details of this procedure are presented in Section 4.

**Figure 1:**
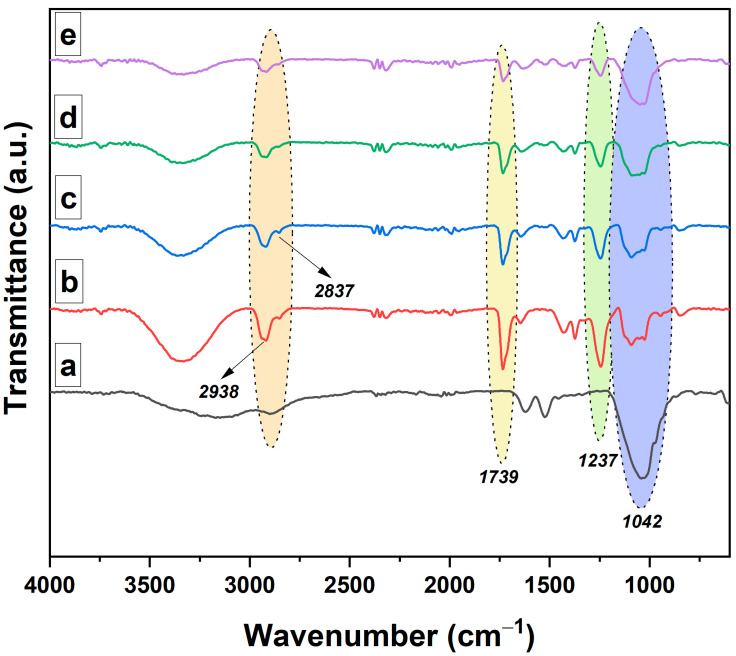
FTIR spectra of the neomycin sulfate (**a**) PVA/XG-80/20 hydrogel (**b**) and the PVA/XG-80/20 hydrogels loaded with neomycin; PVA/XG-80/20-1 (**c**), PVA/XG-80/20-2 (**d**), PVA/XG-80/20-3 (**e**).

The FTIR spectra of the investigated samples are presented in Figure 1. The FTIR absorption spectrum of neomycin sulfate typically exhibits several characteristic absorption bands. Some commonly observed absorption bands for neomycin sulfate are as follows: strong and broad absorption band around 3200–3600 cm^–1^, attributed to the stretching vibrations of O–H (hydroxyl) and N–H (amide) groups, asymmetric and symmetric stretching vibration around 2920–2980 cm^–1^, which corresponds to the C–H (aliphatic) bonds, absorption band around 1650–1700 cm^–1^ (stretching vibrations of C=O (carbonyl) bonds in the amide functional groups) and absorption band around 1450–1500 cm^–1^ (bending vibrations of N–H (amine) groups). For the PVA/XG-80/20 hydrogel, the FTIR spectrum revealed a strong and broad absorption band around 3200–3600 cm^–1^ (stretching vibrations of O–H (hydroxyl) and N-H (amide) groups), indicating the presence of hydroxyl functional groups, and other characteristic bands at 2900–3000 cm^–1^ (asymmetric and symmetric stretching vibrations of C–H (aliphatic) bonds), 1720–1750 cm^−1^ (stretching vibrations of C=O (carbonyl) bonds in PVA), 1600–1650 cm^–1^ (stretching vibrations of C=O (carbonyl) bonds), 1000–1200 cm^–1^ (stretching vibrations of C–O (ether) and C–O–C (glycosidic) bonds) [35]. The inclusion of the bioactive principle in the PVA/XG-80/20 hydrogels was confirmed by the appearance of the following signals in the FTIR-ATR spectra: characteristic absorption bands of stretching vibrations of O–H (hydroxyl) and N–H (amide) groups at 3459 cm^–1^, characteristic absorption bands of the aliphatic C–H bond (asymmetric and symmetric stretching vibration) at 2938 cm^–1^ and 2837 cm^–1^, characteristic bands of the asymmetric and symmetric stretching vibrations of the COO^–^ bond at 1621 cm^–1^ and 1522 cm^–1^, respectively. Other characteristic bands for PVA/XG-80/20 hydrogels loaded with neomycin were located at 1237 cm^–1^ (deformation vibrations of the O–H bond), and at 1042 cm^–1^ associated with the symmetric stretching vibrations of the C–O groups.

### 2.2. Morphological Investigation

**Figure 2:**
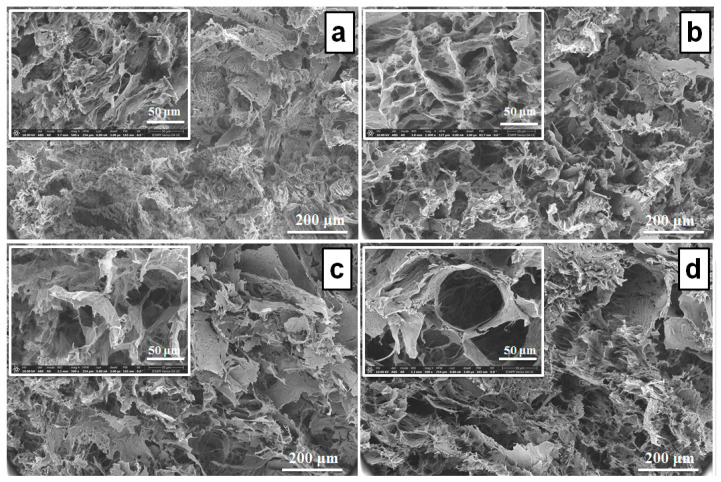
SEM images of the PVA/XG-80/20 hydrogel (**a**) and the PVA/XG-80/20 hydrogels loaded with neomycine; PVA/XG-80/20-1 (**b**), PVA/XG-80/20-2 (**c**), PVA/XG-80/20-3 (**d**).

The cross-sectional microstructure of the PVA/XG-80/20 hydrogels and PVA/XG-80/20 hydrogels loaded with neomycin was analyzed using SEM. The porous structures observed in Figure 2 are a result of the lyophilization process, which involves freeze-drying the hydrogels with a significant water content. Morphological analysis exhibited that all the investigated samples present a typical asymmetric structure. In the case of the samples loaded with neomycin was observed some repression in the formation of the macropores. Increasing the concentration of neomycin sulfate leads to a noticeable increase in the surface wrinkles of the hydrogel. This effect can be attributed to the phenomenon of increased specific surface area within the PVA/XG-80/20 hydrogel network [36]. As a result, the PVA/XG-80/20 hydrogel exhibits a porous structure that allows small molecules like water to easily permeate and reside within these pores. This permeability enables efficient absorption and release of water molecules, contributing to the hydrogel’s increased elasticity [33,37]. During a gradual thawing process [38,39,40], crystalline aggregates form PVA-PVA junctions that serve as anchor points, establishing a robust network. The amorphous domains of the polymer (PVA and XG long chains) play a crucial role in maintaining connectivity among the pores in the physical network. Additionally, the compressive property of the hydrogel is closely associated with its dense skeleton structure.

**Figure 3:**
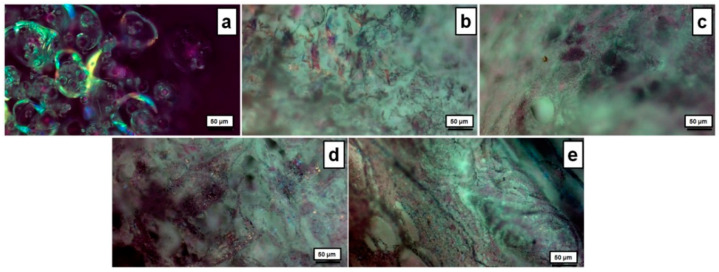
POM images for the investigated samples: neomycin sulfate (**a**), PVA/XG-80/20 (**b**), PVA/XG-80/20-1 (**c**), PVA/XG-80/20-2 (**d**), PVA/XG-80/20-3 (**e**).

The morphology of the microstructure was further examined by means of polarized optical microscopy (POM). As observed in Figure 3a, neomycin sulfate exhibits birefringence under the POM observation, due to its chiral structure and anisotropic crystal lattice [41], which cause the rotation of plane-polarized light passing through the crystals, resulting in characteristic interference patterns and colors. The neat PVA/XG-80/20 sample without any drugs (Figure 3b) exhibited a compact and dense morphology, in contrast to the surfaces of samples containing higher concentrations of neomycin sulfate (Figure 3c–e), which displayed a grainy texture. This grainy texture suggests the potential presence of larger diameter pores within the inner layers of the material. These distinctive colors and birefringency, intensifying with increasing amounts of neomycin sulfate, were also observed in the neomycin-loaded PVA/XG-80/20-1, PVA/XG-80/20-2, and PVA/XG-80/20-3 samples (Figure 3c–e) under POM observation. These observations are indicative of the relatively uniform dispersion of neomycin sulfate within the dry hydrogel network.

### 2.3. Mechanical Investigation

**Figure 4:**
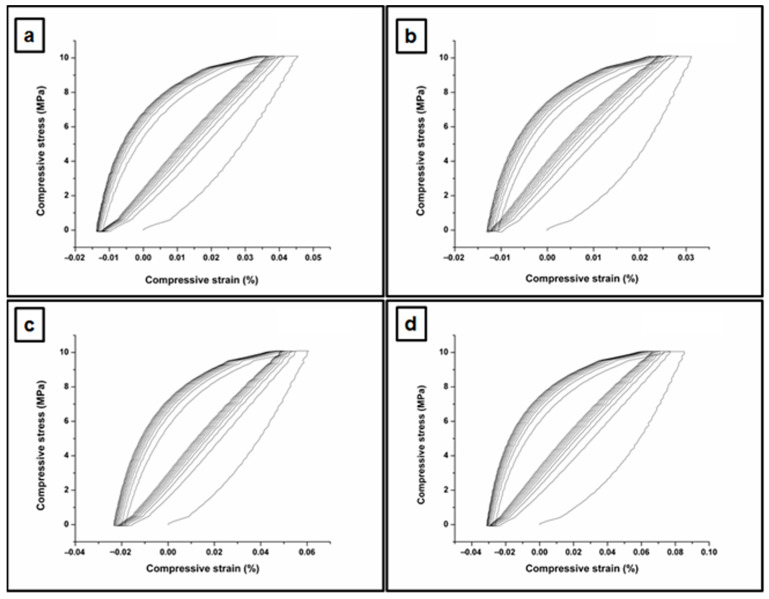
Compressive stress–strain curves of the studied PVA/XG-80/20 hydrogels in the series under 10% compression at room temperature; PVA/XG-80/20 (**a**), PVA/XG-80/20-1 (**b**), PVA/XG-80/20-2 (**c**), PVA/XG-80/20-3 (**d**).

The compression behavior of the hydrogels was characterized by calculating the average testing value across all ten loading–unloading cycles, either for a 10% or 30% compression of the hydrogels (Table 1, Figures S1–S3, and Figure 4, respectively). The dry hydrogels were subjected to swelling in distilled water (PBS solution of 7.4) for three days, but the swollen samples could not be measured. Figure 4 and Figure S3 represent the compressive stress–strain curves of the studied PVA/XG-80/20 hydrogels in the series under 10% compression at room temperature. Hydrogels are typically known for their soft and elastic nature, which allows them to absorb and retain large amounts of water. As a result, they generally exhibit lower mechanical strength compared to traditional solid materials [42,43]. However, the compressive stress that hydrogels can endure can still be significant, especially when designed with appropriate cross-linking and reinforcement strategies. In our case, the values of maximum compressive stress are compared with the values reported in the literature for the hydrogels-based PVA [44]. Figures S1 and S2 show the compression loading–unloading cycles of the neat PVA/XG-80/20 hydrogel and the neomycin sulfate-loaded PVA/XG-80/20 hydrogels, under 30% compression and 10% at room temperature. The hydrogels in the current series displayed a minor hysteresis, indicating good elasticity and bounce-back ability. Importantly, no substantial decrease in the measured compressive stress was observed throughout the 10 loading–unloading cycles (Table 1), implying that the PVA/XG-80/20 hydrogels hold promise for extended use under ambiental conditions. Upon the addition of a smaller amount of neomycin sulfate, the mechanical characteristics of the PVA/XG-80/20 hydrogel were slightly diminished. However, increasing the quantity of neomycin sulfate in PVA/XG-80/20-2 and PVA/XG-80/20-3 formulation demonstrated a favorable cycling behavior, exhibiting a removal efficiency exceeding 80% after five cycles. This indicates a highly effective cycling adsorption performance, with the maximum compressive stress doubling its value in the case of the PVA/XG-80/20-2 and PVA/XG-80/20-3 formulations in comparison with the drug-free sample PVA/XG-80/20. 

The mechanical properties of the neomycin sulfate-loaded PVA/XG-80/20 hydrogels were evaluated to demonstrate their suitability for use in soft tissue applications. 

### 2.4. Swelling Behavior

**Figure 5:**
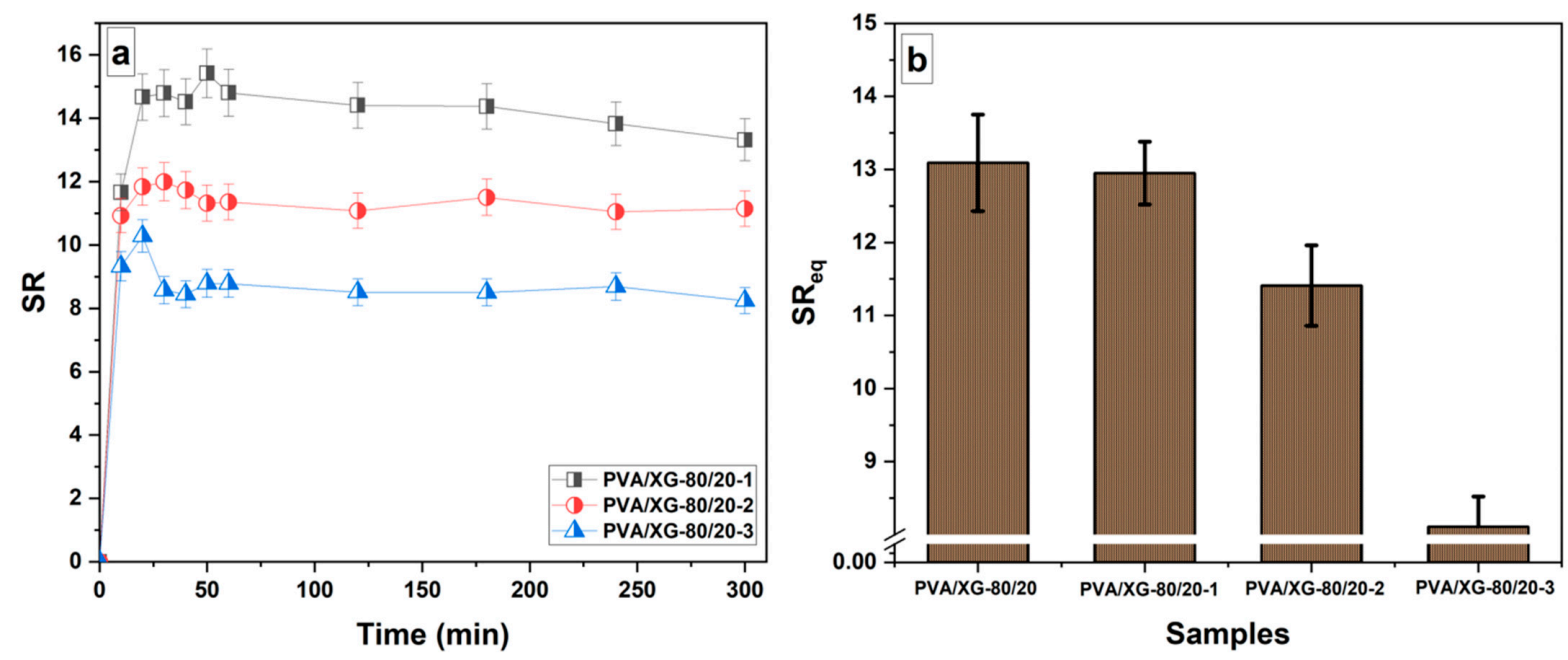
Swelling profiles in phosphate buffer pH 7.4 at 37 °C of PVA/XG-80/20 hydrogels loaded with neomycin sulfate (**a**) and swelling profile at equilibrium for all the samples (**b**).

Understanding the swelling behavior of hydrogels is crucial for their applications, as it affects their mechanical properties, drug release kinetics, and overall performance. By studying the swelling characteristics, researchers can optimize the design of hydrogels for specific applications, such as controlled drug delivery systems or tissue-engineering scaffolds [45]. Figure 5 depicts the swelling behavior of a PVA/XG-80/20 hydrogel incorporated with neomycin sulfate in a phosphate buffer solution with a pH of 7.4, at a temperature of 37 °C. The swelling kinetics exhibit a consistent pattern as observed from the graph. The swelling behavior of the neomycin sulfate-loaded PVA/XG-80/20 hydrogel in the buffer solution undergoes two distinct phases of swelling: an initial rapid increase in swelling followed by a slower swelling process until it reaches its equilibrium swelling capacity. During the early immersion period, the hydrogel experiences a rapid increase in swelling. This could be attributed to the hydrogel’s ability to quickly absorb water from the surrounding buffer solution due to its porous structure. The presence of neomycin sulfate within the hydrogel may also contribute to the initial swelling by attracting water molecules through osmosis or other mechanisms [46]. After the initial rapid swelling, the hydrogel enters a second phase where the swelling process slows down. This indicates that water uptake becomes less pronounced as the hydrogel approaches its equilibrium state. The equilibrium swelling capacity refers to the point at which the hydrogel has absorbed the maximum amount of water it can hold under the given conditions. In this case, it takes approximately 60 min for the hydrogel to reach its equilibrium swelling capacity, which aligns with previously reported findings for PVA/XG-80/20 hydrogels [19]. This indicates that the incorporation of neomycin sulfate influences the swelling profile of the hydrogel. The equilibrium swelling capacity, SR_eq_, exhibits high values for the PVA/XG-80/20 hydrogel loaded with neomycin sulfate. Furthermore, it is observed that the equilibrium swelling capacity decreases with an increase in the content of neomycin sulfate, as depicted in Figure 4. The decrease in swelling degree with an increasing amount of neomycin sulfate loaded into the PVA/XG-80/20 hydrogel can be attributed to several factors. So, loading a higher amount of neomycin sulfate into the PVA/XG-80/20 hydrogel may lead to increased cross-linking between polymer chains. Also, it is possible that the neomycin sulfate molecules may interact with the polymer chains, altering their conformation and restricting their ability to absorb water. Another factor can be correlated with the higher neomycin sulfate loading, which can increase the viscosity of the system, making it more resistant to water penetration. The presence of a larger amount of drug molecules within the system can physically obstruct the movement of water molecules, limiting their access to the polymer matrix. This can hinder swelling and reduce the overall swelling degree. Within the PVA/XG-80/20 hydrogel, neomycin sulfate demonstrates hydrophobic properties, allowing it to adsorb a limited amount of water (PVA/XG-80/20-3). Simultaneously, the hydrogel’s interconnected pores contribute significantly to its ability to retain water effectively and promote a relatively low swelling rate. This can be observed in Figure 2, as depicted in the scanning electron microscopy (SEM) image, where the small volume of interconnected pores within the hydrogel plays a crucial role in facilitating water retention and contributing to the observed modest swelling rate.

### 2.5. In Vitro Release Behavior of Neomycin Drug—Release Kinetics

**Figure 6:**
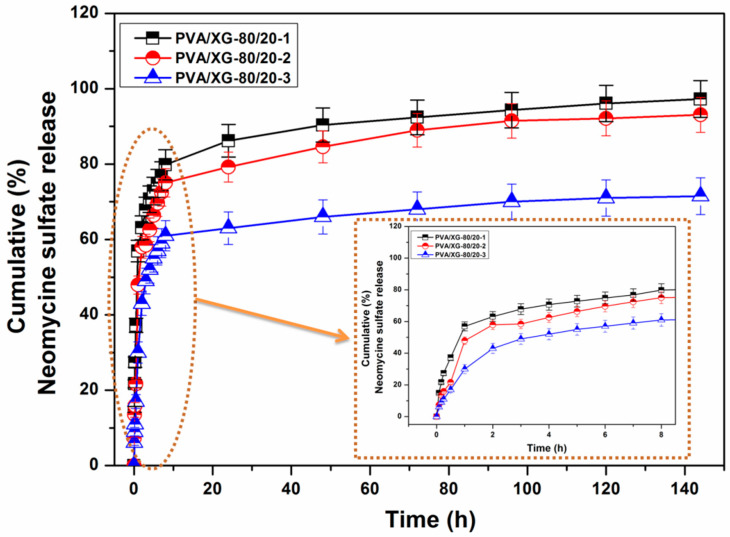
The cumulative % of neomycin released over 4–6 days at 37 °C.

The main objective of this study was to assess the hydrogels’ capability to serve as matrices for the controlled release of neomycin sulfate. To examine this property, in vitro release profiles were measured by placing the hydrogels in PBS at 37 °C for 6 days, and the results were depicted in Figure 6. The release of the encapsulated drug occurred in three distinct stages: Firstly, there was a burst effect during the initial 1–2 h, with approximately 57% of the drug released into the PBS medium. Subsequently, a slower release took place over the following 8 h, resulting in the release of 79% of the drug. Finally, there was a continuous, gradual release over the next 6 days, leading to almost complete passage of the neomycin into the PBS medium. It is known that the in vitro release rate and mechanism profile of the drug are complex processes, being controlled on multiple factors such as: swelling of the matrix, diffusion of the drug, chain relaxation, and erosion rate of the matrix, and they are also dependent on the interaction between the encapsulated drug and the matrix [47].

Comparing the release rates of the three studied formulations, the influence of the neomycin content, which was observed on the morphology (dimensions of the pore size) and swelling capacity and the physical interaction between the drug and the matrix, could be observed. The samples with the highest swelling capacity (PVA/XG-80/20-1) exhibited the highest release rate due to the increased ease of drug dissolution and diffusion into the release medium. Also, the PVA/XG-80/20-1 sample showed a slightly faster release than the PVA/XG-80/20-2, probably because the weak interaction between matrix and drug led to a higher release rate. In the case of the sample with the highest content of the drug (PVA/XG-80/20-3), was observed a slowest release rate, according to the increase of the intra- and inter-molecular interactions between the matrix and the drug, which is in close correlation with other studies [48].

Additionally, to gain a more comprehensive understanding of the neomycin drug-release mechanism from the tested formulations, a mathematical analysis of the in vitro release profile was conducted (Table 2). Five distinct mathematical models were applied to fit the in vitro release data for each of the three stages (Figure S4) [49,50].

The graphical representation of the equations for all five mathematical models demonstrated a strong correlation (R^2^ = 0.943–0.999) with the data gathered during the initial stage of the in vitro release experiment, across all three samples. It can be said that the mechanism of the neomycin release in the first stage, is complex, being governed by many factors, as detailed further. The fitting of the Zero Order model, in the first stage, reflect both the influence of the dissolution of the drug as a function of time and the fast swelling of the matrix, leading to a faster dissolution. The lower release constant of the formulation with a higher content of the drug, (PVA/XG-80/20-3) also reflects the influence of the strength of the drug anchoring into the matrix. The increase of the physical forces between the drug and the hydrogel matrix prevented its fast dissolution and promoted its slow diffusion to the release medium. The Higuchi model (R^2^ = 0.960–0.996) shows that the diffusion of the drug molecules through the matrix also plays an important role in the drug release process. The higher constant proportionality in the case of the samples with a lower amount of the drug shows an easier diffusion of the neomycin through the matrix. The good fitting of the Hixson–Crowell model (R^2^ = 0.969–0.995) confirmed that the matrix erosion by biodegradation also has an important role on the neomycin diffusion through the matrix. The influence of the hydrogel matrix on the neomycin diffusion was confirmed by good fitting of the Korsmeyer–Peppas model (R^2^ = 0.975–0.998), which provided important information about to the type of diffusion. All the samples provided the exponent n value from 0.53 to 0.66, characteristic for a non-Fickian anomalous transport phenomenon controlled by both drug diffusion through the matrix and matrix swelling. The good fitting of the First Order model (R^2^ = 0.94–0.997) indicates that the neomycin released is also dependent on the amount encapsulated into the hydrogel matrix [50].

In the second and third stage a good fitting of all five mathematical models was observed. Compared to the first stage, a decrease of the proportionality constants and almost an equalization of their values attributed to a slower and same release rate of the neomycin through the matrix was observed. An important change was seen for the diffusional coefficient n with a drastic decrease to the range 0.16–0.22 (second stage) and 0.07–0.09 (third stage), attributed to the change of the neomycin diffusion to a pseudo-Fickian pattern with slow drug release. In this stage, it seems that the morphological changes of the hydrogels matrix (swollen or erosion of the pore walls) increase the diffusion of the drug molecules and, thus, the dissolution of the remaining drug molecules’ strong anchoring into the matrix, slowing down. 

Based on the data, it can be inferred that the neomycin release mechanism was predominantly influenced by the drug’s state within the matrix, which in turn was controlled by the density of intra- or inter-molecular interactions between the drug and the matrix. For samples with a lower amount of the drug, the density of these interactions was lower, resulting in less anchoring of the drug molecules within the hydrogel matrix. This led to faster dissolution during the initial stage of release, followed by a gradual and sustained release over the course of 6 days. Conversely, in the case of the sample with the highest drug amount (PVA/XG-80/20-3), the increased interactions between the drug and the matrix resulted in stronger anchoring through physical forces. As a consequence, the release of neomycin in the initial stage was delayed or significantly reduced, leading to a lower total amount of drug delivered. Throughout the in vitro release experiments, all formulations exhibited stability under the experimental conditions.

### 2.6. Antimicrobial Activity of Neomycin Sulfate-Loaded PVA/XG-80/20 Hydrogels 

For the antimicrobial test, the samples of PVA/XG-80/20 and PVA/XG-80/20 hydrogels loaded with neomycin were depicted dimensioned (10 mm), but on the surface of the culture medium, the matrices changed their morphology so that in order to ensure a uniform interpretation of the results, the width of the zone of inhibition of the microbial culture bounded by the edge of the sample and the rim was measured. The arithmetic mean of the values obtained in triplicate and the standard deviation of the mean were calculated (Table 3). The results obtained show that the mean values of the samples follow a normal distribution from a statistical point of view.

Testing using the diffusimetric method shows that the PVA/XG control sample has no antimicrobial activity against any of the strains tested, so the antimicrobial activity of the PVA/XG-80/20-1, PVA/XG-80/20-2, and PVA/XG-80/20-3 samples is determined by the concentration of neomycin sulphate.

Both Gram-positive and Gram-negative species were found to be sensitive to all samples tested (graphical representation).

**Figure 7:**
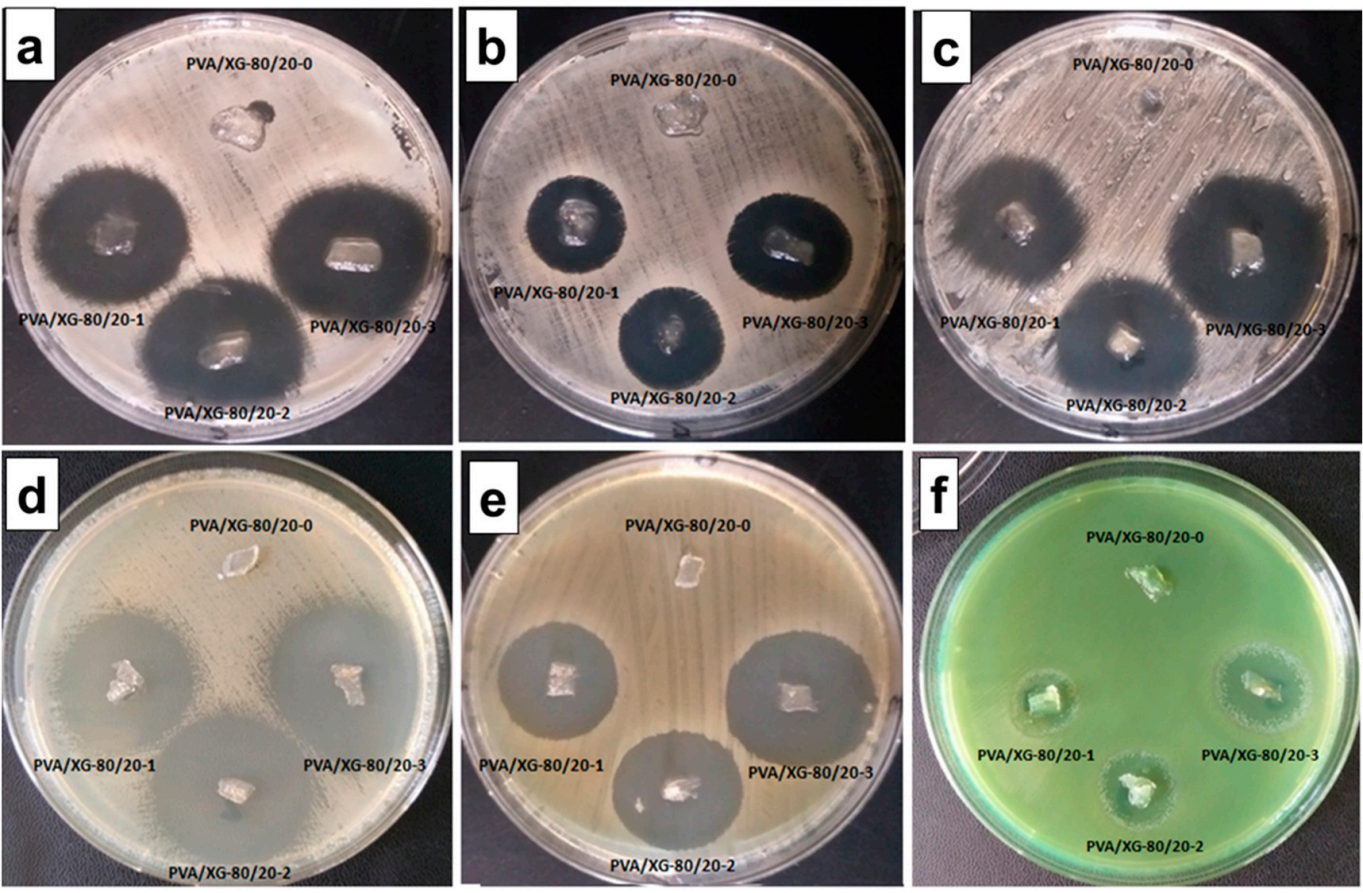
Antimicrobial activity of *Staphylococcus aureus* ATCC 25923 (**a**), *MRSA* ATCC 33591 (**b**), *Staphylococcus epidermidis* ATCC 12228 (**c**), *Escherichia coli* ATCC 25922 (**d**), *Klebsiella pneumoniae* ATCC 13883 (**e**), and *Pseudomonas aeruginosa* ATCC 27853 (**f**).

It can be seen that against the species *Staphylococcus aureus* ATCC 25923, the microbial zone of inhibition varied between 9.33 ± 0.577 mm (PVA/XG-80/20-1) and 12.66 ± 0.577 mm (PVA/XG-80/20-3) (Figure 7a). Against *MRSA* ATCC 33591, zones of inhibition averaged between 5.66 ± 0.577 mm (PVA/XG-80/20-1) and 10.66 ± 0.577 mm (PVA/XG-80/20-3) (Figure 7b). Against *Staphylococcus epidermidis* ATCC 12228, the range of inhibition zone values was between 11 ± 0.577 mm (PVA/XG-80/20-1) and 15.33 ± 0.577 mm (PVA/XG-80/20-3) (Figure 7c).

The cut-off values of the mean inhibition values obtained with *Escherichia coli* ATCC 25922 ranged from 9.66 ± 0.577 mm (PVA/XG-80/20-1) to 13.66 ± 0.577 mm (PVA/XG-80/20-3) (Figure 7d). Against *Klebsiella pneumoniae* ATCC 13883 the zones of inhibition varied between 9.66 ± 0.577 mm (PVA/XG-80/20-1) and 11.66 ± 0.577 mm (PVA/XG-80/20-3) (Figure 7e). Against *Pseudomonas aeruginosa* ATCC 27853, the antimicrobial effect was lower, and the value limits of the inhibition zones varied between 2.66 ± 0.577 mm (PVA/XG-80/20-1) and 6.66 ± 0.577 mm (PVA/XG-80/20-3) (Figure 7f).

The results show that the antimicrobial activity spectrum correlates with the amount of antibiotic contained in the test sample. All antibiotic concentrations added to the PVA/XG matrices are active against all bacterial strains tested. Neomycin is a natural hydrophilic aminoglycoside isolated mainly from *Streptomyces* sp. It is known for its bacteriostatic and bactericidal activity against Gram-positive and Gram-negative bacteria [51].

The mode of action of neomycin is based on the inhibition of bacterial protein synthesis, resulting in cell death. The mechanism is known and is based on its ability to inhibit bacterial ribosomes by binding to the ribosomal subunit 30S of some bacteria and interrupting translation for bacterial protein synthesis [52,53]. The bacterial translation is initiated by the normal binding of mRNA to the 30S ribosomal subunit and subsequent binding to the 50S subunit for elongation [53].

## 3. Conclusions

In summary, this research focuses on the utilization of biocompatible polymers (PVA and XG) to fabricate hydrogel networks through cross-linking with oxalic acid using the freezing/thawing method. The incorporation of neomycin sulfate aims to impart antimicrobial properties to the hydrogels, paving the way for the development of potential low-class topical antibacterial formulations. 

The incorporation of higher amounts of neomycin sulfate in the PVA/XG-80/20-2 and PVA/XG-80/20-3 formulations showed promising cycling characteristics. These formulations exhibited a removal efficiency of over 80% after five cycles, indicating a remarkably effective adsorption performance during repeated use. Furthermore, when compared to the drug-free sample PVA/XG-80/20, both the PVA/XG-80/20-2 and PVA/XG-80/20-3 formulations demonstrated a significant increase in maximum compressive stress.

The antimicrobial activity of neomycin on a broad spectrum of microorganisms, even those resistant to antibiotics as demonstrated in our study (*MRSA* ATCC 33591), and as it is known to have the lowest toxicity in vivo, makes this antibiotic an excellent candidate for topical therapy.

## 4. Materials and Methods

### 4.1. Materials

Poly(vinyl alcohol) (MW 9000~10,000, 87–89% hydrolyzed) and oxalic acid were acquired from Merch (Darmstadt, Germany). The Xanthan gum transparent grade was purchased from Elements (Vienna, Austria). Neomycin sulfate was obtained from Yichang Sanxia Pharmaceutical Co., Ltd., Yichang, China. All other reagents were either directly used from commercial sources or purified using standard methods.

### 4.2. Preparation of PVA/XG-20/80 Hydrogels

The PVA/XG-80/20 hydrogel was prepared using the following procedure: First, 0.4 g of PVA and 0.1 g of xanthan gum were dissolved in an appropriate amount of double-distilled water (8%) and mixed thoroughly with continuous stirring at room temperature. Then, 0.08 g of oxalic acid was added to the mixture, and stirring was continued until a homogeneous blend was achieved. Next, the resulting mixture was subjected to a freeze/thaw process for 5 days. Afterward, the PVA/XG-80/20 hydrogels were dried using the lyophilization method. The hydrogels were placed in an oven and kept at 100 °C for 1 h to complete the drying process. The obtained sample was subjected to the dialysis process to remove the residual agent of cross-linking. This stage is very important to obtain materials that correspond to the purity requirements imposed by the use in the biomedical field or pharmaceutical. Also, the biomaterial must be chemically inert or have a therapeutic effect; therefore, special attention is paid to minimizing the effect cytotoxicity of residual oxalic acid. The samples were finnaly dried by lyophilization.

### 4.3. Preparation of PVA/XG-20/80 Hydrogels Loaded with Neomycin Sulfate

The inclusion of the active principle (neomycin sulfate) in the PVA/XG-80/20 hydrogel, at a temperature of 37 °C, in a phosphate buffer solution of pH 7.4, was achieved using the maximum of the amount of solvent absorbed by the support, evaluated from the swelling curve, with evaporation its subsequent and sample drying by lyophilization. In Table 4 are listed the wt.% of neomycin loaded into the PVA/XG-80/20 hydrogel.

Encapsulation efficiency is a crucial parameter when designing drug delivery systems or formulations, as it indicates the percentage of the desired substance that is successfully encapsulated within the carrier. The data reported in Table 4 confirmed higher encapsulation efficiency in the case of the PVA/XG-80/20-2 and PVA/XG-80/20-3 samples, indicating that a large proportion of neomycin was successfully trapped inside the carrier hydrogel matrix, which is desirable for efficient and controlled delivery of the encapsulated substance. The results obtained for drug loading percentage indicate that a significant amount of the drug is present in the carrier system, which can be beneficial for achieving a potent therapeutic effect. However, higher drug loading may also lead to challenges such as physical stability or burst release of the drug. Thus, balancing drug loading with the stability and controlled release of the drug is crucial to ensure the efficacy and safety of the drug delivery system. 

### 4.4. Methods

#### 4.4.1. FTIR Analysis

Scans of the PVA/XG hydrogels were recorded using a LUMOS Microscope Fourier Transform Infrared (FTIR) spectrophotometer (Bruker Optik GmbH, Ettlingen, Germany). The instrument was equipped with an attenuated total reflection (ATR) device, and the scans were taken within the range of 4000 to 500 cm^–1^ with a resolution of 4 cm^–1^.

#### 4.4.2. Scanning Electron Microscopy

Scanning electron microscopy (SEM (FEI Company, Brno, Czech Republic)) was performed on a Quanta 200 instrument, at 30 kV, with a magnification of 380–3600. During the experimental investigations, small, approximately 5 mm sized sections of the PVA/XG hydrogel and PVA/XG hydrogel loaded with neomycin sulfate were affixed to an aluminum specimen holder using conductive adhesive tape.

#### 4.4.3. POM Analysis

The morphology of the studied samples was examined by capturing microphotographs using a Zeiss Microscope Axio Imager A2M with a 10× objective (Carl Zeiss Microscopy GmbH, Oberkohen, Germany) at room temperature. The drug was examined in a powder state while the hydrogel networks in their dry state were cut into small pieces and placed on glass plates for examination, with the microscopic view set at the edge of the material before taking photos of each sample in the series. The examination aimed to provide further insights into the samples’ structure and morphology.

#### 4.4.4. Mechanical Properties

The compression tests were performed on an Instron 3365 machine equipped with a load cell of 500 N. The tests were made on cylindrical specimens 4 mm in diameter. The compression tests (experiments/analysis) were made in order to discover how the materials behave while being pressed at different compressive strains of 10 and 30%, with a loading speed of 3 mm/min, for ten cycles.

#### 4.4.5. Reversible Swelling Behavior of the Hydrogel 

The swelling and deswelling behavior of the hydrogel was assessed using a conventional gravimetric analysis method. Initially, a set of dry hydrogel samples with a known weight (≈0.05 g (0.5 × 0.5 cm^2^)) were immersed in a PBS = 7.4. The samples were placed in the medium to allow them to swell. After that, the swelled samples were taken out from the petri dish with caution, gently dried using absorbent papers, and weighed at specific time intervals. The time intervals included 10 min, 15 min, 30 min, 40 min, 50 min, 60 min, and subsequently every hour for a total duration of 5 h. For each hydrogel, three randomly selected samples were tested, and the average value was recorded. The swelling ratio (SR) of each hydrogel was calculated using the following equation [54]:

(1) $$SR=\frac{W_{t}-W_{d}}{W_{d}}$$

where W_t_ is the mass of the hydrogel measured after immersion in phosphate buffer solution (PBS) = 7.4 for a time period of t and W_d_ is the dry mass of the sample.

#### 4.4.6. Encapsulation Efficiency and Drug Loading 

The encapsulation efficiency (EE) and drug loading (DL) have been evaluated according to the following equations (Equations (2) and (3)) [55,56]:

(2) $$\mathrm{EE}\;\left(\%\right)=\frac{{\mathrm{Neomycin}\;\mathrm{sulfate}}_{\mathrm{encap}}}{{\mathrm{Neomycin}\;\mathrm{sulfate}}_{\mathrm{total}}}\times 100$$

(3) $$\mathrm{DL}\;\left(\%\right)=\frac{{\mathrm{Neomycin}\;\mathrm{sulfate}}_{\mathrm{encap}}}{\mathrm{PVA}/\mathrm{XG}-80/20_{\mathrm{total}}}\times 100$$

where EE (%) is the encapsulation efficiency, Neomycin sulfate_encap_ is mass of neomycin sulfate encapsulated (g) measured by mass balance; Neomycin sulfate_total_ is the initial Neomycin sulfate_total_ mass in PBS of pH 7.4 (g), DL (%) is drug loading efficiency, and PVA/XG-80/20_total_ is the initial PVA/XG-80/20 mass in PBS of pH 7.4 (g) (Table 4).

#### 4.4.7. Determination of the Neomycin Realized by ^1^H-NMR Spectroscopy

The proton spectra were acquired using a Bruker Avance NEO 400 MHz spectrometer (Bruker Biospin, Ettlingen, Germany), which was equipped with a 5 mm broadband inverse detection z-gradient probe. The NOESY water-presaturation pulse sequence was employed for the recordings. The samples were run in 5 mm Bruker 500 (Boro500) NMR tubes. To the 0.54 mL neomycin solution in PBS, 0.06 mL of 5 mM sodium 3-(trimethylsilyl)-[2,2,3,3-d4]-1-propionate (TSP) (Aldrich) in D_2_O (Aldrich) was added. Before transferring into a NMR tube, the solution was stirred at 500 rpm for 3 min. All the spectra were registered using 64 scans and a relaxation delay of 30 s, in order to obtain quantitative results. The spectra were processed manually using the Bruker TopSpin 4.1.1 software, which serves as the spectrometer control and processing tool. Chemical shifts are presented in δ units (parts per million, ppm) and were referenced to the TSP internal standard, which was set at 0.0 ppm. Recording temperature for water presaturation NMR spectra was 300 K. For the calibration curve, five neomycin solutions in PBS of different concentrations, 0.1, 0.17, 0.3, 0.5, and 1 mg/mL, were prepared. A total of 0.54 mL from these solutions was transferred in the NMR tubes, adding 0.06 mL of 5 mM TSP in D_2_O. The solutions were also stirring at 500 rpm for 3 min. After NMR dilution, theoretical concentrations became 0.09, 0.153, 0.27, 0.45, and 0.9 mg/mL. The experimental concentration of neomycin was obtained using the following expression:

(4) $$C_{\mathrm{Neomycin}}=\frac{9}{1}\times \frac{I_{\mathrm{Neomycin}}}{I_{\mathrm{TSP}}}\times C_{\mathrm{TSP}}$$

were $I_{Neomycin}$ is the integral value of the signal corresponding to neomycin H1′ proton [57,58]; $I_{TSP}$ is the integral value of the signal corresponding to TSP protons and $C_{TSP}$ is the concentration of TSP in NMR solution/is the concentration of TSP after NMR dilution. The calibration curve obtained by the representation of theoretical vs experimental values is presented in Figure S5 For the NMR study of neomycin released from formulations, to 0.54 mL neomycin solution in PBS, 0.06 mL of 5 mM sodium 3-(trimethylsilyl)-[2,2,3,3-d4]-1-propionate (TSP) (Aldrich) in D_2_O (Aldrich) was added. Before transferring into a NMR tube, the solution was stirring at 500 rpm for 3 min. The ^1^H NMR spectra were recorded using the NMR parameters presented above. The neomycin concentrations were calculated from the ^1^H NMR spectra using the same relation as for the calibration curve. 

#### 4.4.8. The In Vitro Release Behavior 

The release behavior of neomycin from the formulations was studied using in vitro methods. The samples, containing a specific amount of the drug, were placed in a thermostatic chamber at a constant temperature of 37 °C. The release was investigated in a saline phosphate buffer (PBS) with a pH of 7.4. The experimental procedure involved immersing the samples into vials containing 10 mL of PBS. At specific time intervals, 2 mL of the supernatant (the liquid portion above the settled particles) was taken out and replaced with 2 mL of fresh PBS. This process was repeated for a duration of 6 days. To determine neomycin concentrations in the collected samples, ^1^H NMR spectra were used, and the concentrations were calculated based on the calibration curve previously generated for neomycin (as detailed in the supporting information, Figure S5). The cumulative (%) neomycin released from the samples was calculated according to the following Equation (5):

(5) $$\%\;\mathrm{Neomycin}=\frac{10C_{n}+2C_{n-1}}{m_{0}}\times 100$$

in which C_n_ and C_n−1_ represent the neomycin concentrations in the extracted supernatant after n and n − 1 withdrawing steps, respectively, while m_0_ represents the amount of neomycin loaded in the formulations. The tests were performed in duplicate and the average value was taken as result. In order to evaluate the mechanism of the drug release, the data were fitted on the equations of the Korsmeyer–Peppas, Zero-order, First-order, Higuchi, and Hixson–Crowell mathematical models [59]. 

#### 4.4.9. The Mathematical Models Used for the Kinetic Data Analysis

*(a)*  Zero-order model: $Q_{t}=Q_{0}+K_{0}\cdot t$, where Q_0_ is the initial quantity of the drug, Q_t_ is the quantity of drug dissolved in at time t and K_0_ is the zero-order release constant;
*(b)*  Higuchi model: $Q_{t}=K_{H}\cdot t^{\frac{1}{2}}$, where Q_t_ is the amount of drug released in the time t and K_H_ is the Higuchi dissolution constant;
*(c)*  Hixson–Crowell model: $W_{0}^{1/3}-W_{t}^{1/3}=K\cdot t$, where W_0_ is the initial amount of drug in the formulation, W_t_ is the remaining amount of drug in the formulation at time t and K is a constant;
*(d)*  Korsmeyer–Peppas model: $\frac{M_{t}}{M_{\infty }}=K\cdot t^{n}$, where M_t_/M_∞_ is the fraction of drug released at the time t, K is the release rate constant and n is the release exponent;
*(e)*  First-order model: ${\mathrm{logQ}}_{t}={\mathrm{logQ}}_{0\;}+K\cdot t/2.303$, where Q_0_ is the initial amount of drug, Q_t_ is the quantity of drug released in the time t and K is the first order release constant.

#### 4.4.10. Antimicrobial Activity

The antimicrobial activity of PVA/XG-80/20 hydrogel and PVA/XG-80/20 loaded with neomycin sulfate was qualitatively determined using the Kirby–Bauer diffusimetric method adapted for this type of biomaterials, according to a method from a previously reported work [33]. For this purpose, standardized bacterial cultures *Staphylococcus aureus* ATCC 25923, *Methicillin-resistant Staphylococcus aureus (MRSA) ATCC 43300, Staphylococcus epidermidis ATCC 12228* (Gram-positive) and *Escherichia coli ATCC 25922*, *Pseudomonas aeruginosa ATCC* 27853, and *Klebsiella pneumoniae ATCC 13883* (Gram-negative) were brought to a cell density corresponding to the 0.5 McFarland turbidity standard (1.5 × 10^8^ cfu/mL). The microbial suspensions were seeded on the surface of the Muller–Hinton Agar (Oxoid) solid culture medium, after which the PVA/XG-80/20 hydrogel and PVA/XG-80/20 loaded with neomycin sulfate (0.05 g) were distributed. After 24 h of incubation, the width of the microbial zone of inhibition of each monitored sample was measured. Samples were tested in triplicate for which the mean and standard deviation (SD) were calculated. The standard deviation is a statistical indicator that shows the variation in the results of each tested sample compared to the average obtained.

## Figures and Tables

**Table 1:** The maximum value of the compressive stress (MPa) for the measured dry hydrogels.

Sample	Maximum Compressive Stress (MPa)	
	10%	30%
PVA/XG-80/20	0.039 ± 0.003	0.116 ± 0.005
PVA/XG-80/20-1	0.026 ± 0.002	0.089 ± 0.004
PVA/XG-80/20-2	0.052 ± 0.004	0.202 ± 0.010
PVA/XG-80/20-3	0.071 ± 0.006	0.212 ± 0.011

**Table 2:** The kinetic data fitted on different mathematical models for: the first stage (5 min–1 h), the second stage (3–8 h), and the third stage (1–6 days).

	Model	Zero Order		First Order		Higuchi		Korsmeyer–Peppas			Hixson–Crowell	
Code												
First stage		R^2^	K_0_	R^2^	K	R^2^	K_H_	R^2^	K	n	R^2^	K
PVA/GX-80/20-1		0.985	44	0.997	0.72	0.996	58	0.998	55	0.53	0.995	−0.94
PVA/GX-80/20-2		0.978	42	0.943	0.42	0.96	48	0.975	40	0.66	0.969	−0.83
PVA/GX-80/20-3		0.973	19	0.99	0.26	0.993	34	0.991	28	0.63	0.989	−0.37
Second stage		R^2^	K_0_	R^2^	K	R^2^	K_H_	R^2^	K	n	R^2^	K
PVA/GX-80/20-1		0.98	2.6	0.989	0.09	0.991	11	0.993	56	0.16	0.988	−0.09
PVA/GX-80/20-2		0.992	3.3	0.983	0.09	0.999	15	0.999	44	0.26	0.998	−0.1
PVA/GX-80/20-3		0.989	2.4	0.995	0.05	0.998	11	0.999	38	0.22	0.994	−0.06
Third stage		R^2^	K_0_	R^2^	K	R^2^	K_H_	R^2^	K	n	R^2^	K
PVA/GX-80/20-1		0.96	0.09	0.995	0.01	0.991	1.5	0.998	69	0.07	0.995	−0.008
PVA/GX-80/20-2		0.900	0.08	0.948	0.009	0.95	1.9	0.983	59	0.09	0.930	−0.007
PVA/GX-80/20-3		0.989	0.09	0.994	0.003	0.999	1.4	0.993	49	0.07	0.993	−0.003

R^2^: correlation coefficient; K: proportionality constant; n: release exponent.

**Table 3:** The mean value and standard deviation (SD) of the inhibition zones obtained to antimicrobial testing against bacteria strains.

	*Staphylococcus aureus* *ATCC 25923*	*Methicillin-resistant Staphylococcus aureus (MRSA) ATCC 33591*	*Staphylococcus epidermidis ATCC 12228*	*Escherichia coli* *ATCC 25922*	*Klebsiella pneumoniae ATCC 13883*	*Pseudomonas* *aeruginosa ATCC 27853*
**PVA/XG-80/20**	0	0	0	0	0	0
**PVA/XG-80/20-1**	9.33 ± 0.577	5.66 ± 0.577	11 ± 0.577	9.66 ± 0.577	9.66 ± 0.577	2.66 ± 0.577
**PVA/XG-80/20-2**	11.33 ± 0.577	9.66 ± 0.577	11.66 ± 0.577	12	10	4.66 ± 0.577
**PVA/XG-80/20-3**	12.66 ± 0.577	10.66 ± 0.577	15.33 ± 0.577	13.66 ± 0.577	11.66 ± 0.577	6.66 ± 0.577

**Table 4:** The composition of the PVA/XG-80/20 hydrogel and the amount (g/wt.%) of neomycin sulfate loaded into the PVA/XG-80/20 hydrogel.

Samples	PVA (g)	Xanthan Gum (g)	Oxalic Acid (g)	Distilled Water (mL)/(%)	Amount of Neomycin Sulfate (g)	EE ^a^ (%)	DL ^b^ (%)
**PVA/XG-80/20**	0.4	0.1	0.08	7.25/8	-	-	-
**PVA/XG-80/20-1**	0.4	0.1	0.08	7.25/8	0.0176	8.74	2.5
**PVA/XG-80/20-2**	0.4	0.1	0.08	7.25/8	0.0928	23	11.6
**PVA/XG-80/20-3**	0.4	0.1	0.08	7.25/8	0.211	34.99	18.7

^a^ encapsulation efficiency (EE) and ^b^ drug loading (DL).

## Data Availability

The data that support the findings of the current study are listed within the article.

## Supplementary Materials

The following supporting information can be downloaded at: https://www.mdpi.com/article/10.3390/gels9080655/s1, Figure S1: Compression tests on the studied PVA/XG-80/20 hydrogels in the series under 30% compression at room temperature; PVA/XG-80/20 (**a**), PVA/XG-80/20-1 (**b**), PVA/XG-80/20-2 (**c**), PVA/XG-80/20-3 (**d**); Figure S2. Compression tests on the studied PVA/XG-80/20 hydrogels in the series under 10% compression at room temperature PVA/XG-80/20 (**a**), PVA/XG-80/20-1 (**b**), PVA/XG-80/20-2 (**c**), PVA/XG-80/20-3 (**d**); Figure S3. Compressive stress–strain curves of the studied PVA/XG-80/20 hydrogels in the series under 30% compression at room temperature; PVA/XG-80/20 (**a**), PVA/XG-80/20-1 (**b**), PVA/XG-80/20-2 (**c**), PVA/XG-80/20-3 (**d**); Figure S4. Different mathematical models fitted on the in vitro release profile on each of the three stages; Figure S5. Calibration curve as obtained from NMR data.
