# Supplementary material for: Evaluation of Poly(vinyl alcohol)–Xanthan Gum Hydrogels Loaded with Neomycin Sulfate as Systems for Drug Delivery
Source: Gels. 2023 Aug 14;9(8):655. doi: 10.3390/gels9080655 (PMC10454009; doi:10.3390/gels9080655)
Supplement: Supplementary file 1 [file gels-09-00655-s001.zip › gels-2508125-supplementary.pdf]

Evaluation of PVA-Xanthan Gum Hydrogels Loaded with Neomycin Sulfate as Systems for drug delivery

Diana Serbezeanu[1], Manuela Maria Iftime[1], Gabriela-Liliana Ailiesei[1], Alina-Mirela Ipate[1], Alexandra Nistor[1], Tăchiţă Vlad-Bubulac[1], Cristina Mihaela Rîmbu[2]

[1] Department of Polycondensation and Thermally Stable Polymers, "Petru Poni" Institute of Macromolecular Chemistry, 41A Grigore Ghica Voda Alley, 700487 Iasi, Romania

[2] Department of Public Health, Iasi University of Life Sciences, 8 Mihail Sadoveanu Alley, 707027 Iasi, Romania

* Correspondence: diana.serbezeanu@icmpp.ro

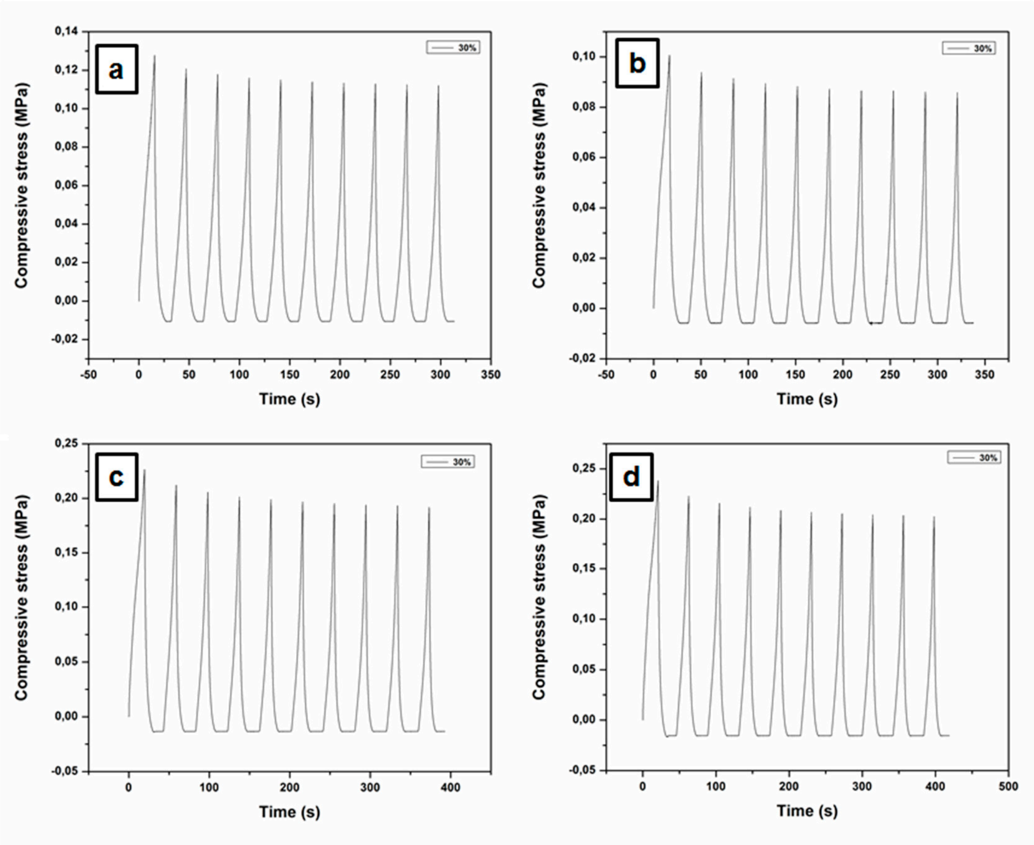

Figure S1. Compression tests on the studied PVA/XG-80/20 hydrogels in the series under 30% compression at room temperature; PVA/XG-80/20 (a), PVA/XG-80/20-1 (b), PVA/XG-80/20-2 (c), PVA/XG-80/20-3 (d).

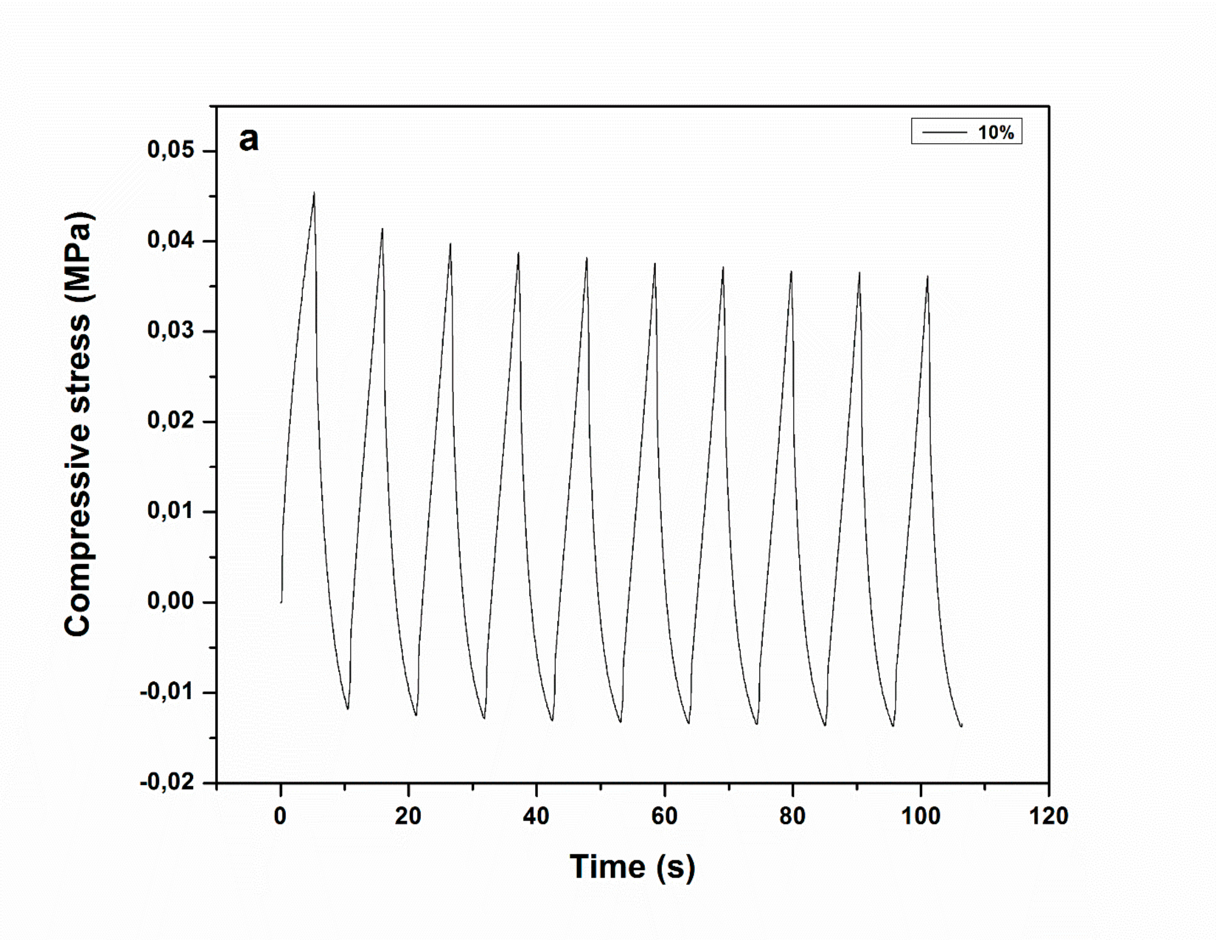

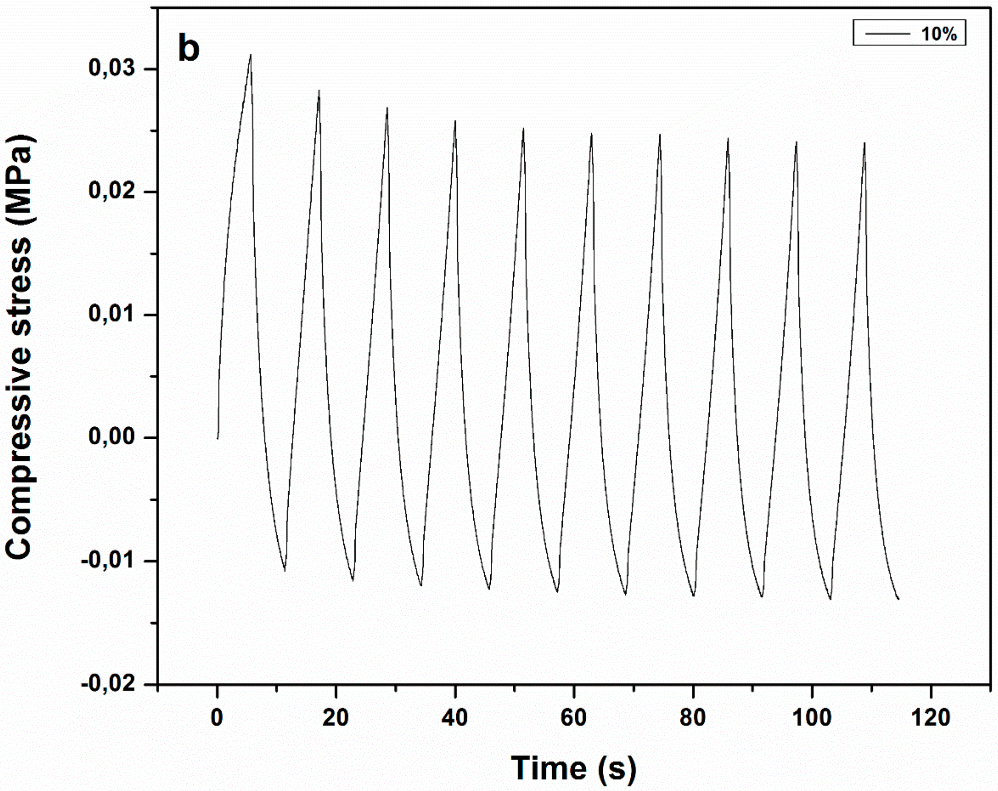
b
10%
Compressive stress (MPa)
0,03
0,02
0,01
0,00
-0,01
-0,02
0
20
40
60
80
100
120
Time (s)

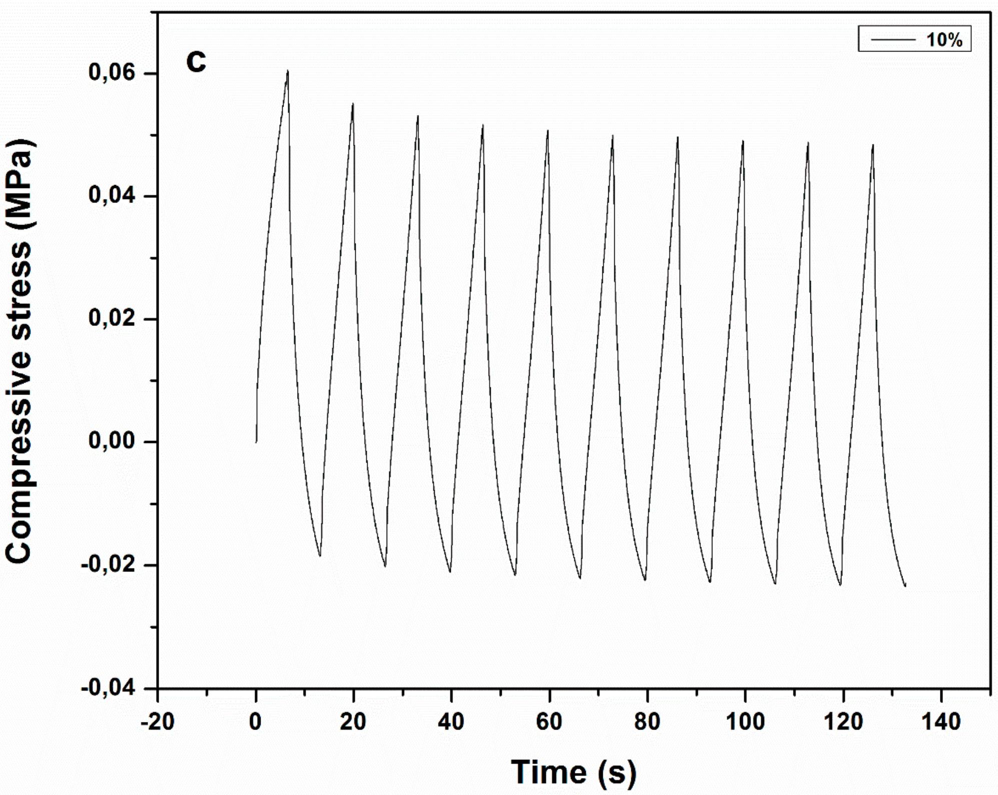
c
10%
0,06
0,04
0,02
0,00
-0,02
-0,04
Compressive stress (MPa)
-20
0
20
40
60
80
100
120
140
Time (s)

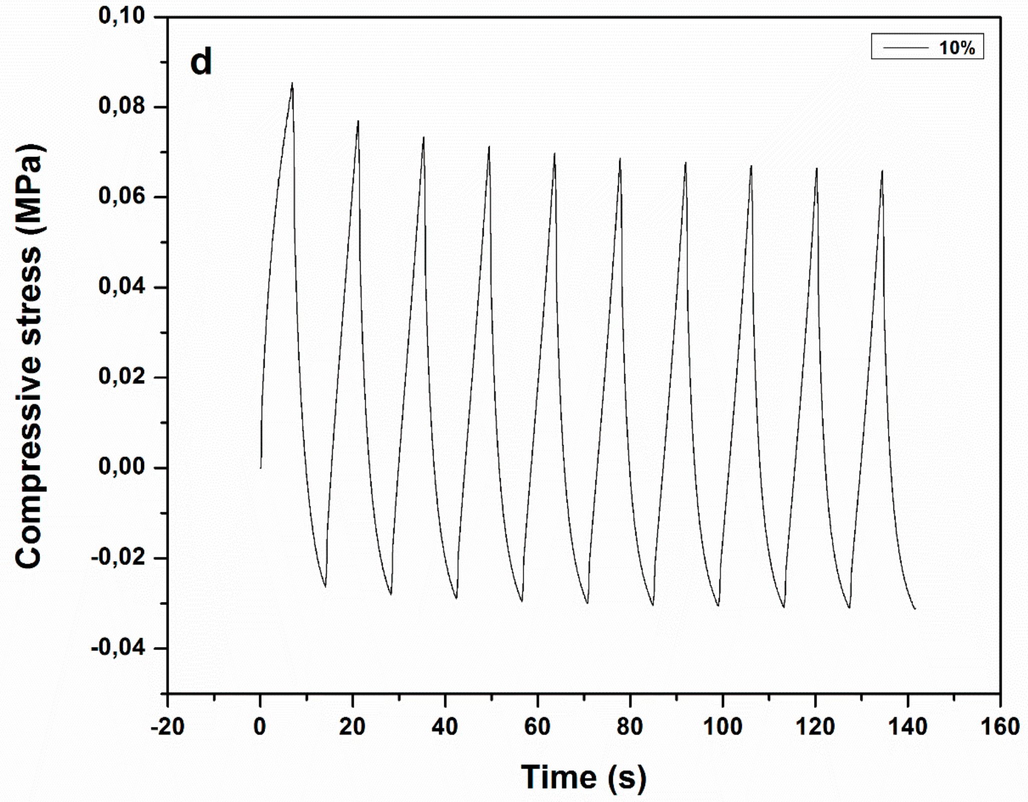

Figure S2. Compression tests on the studied PVA/XG-80/20 hydrogels in the series under 10% compression at room temperature PVA/XG-80/20 (a), PVA/XG-80/20-1 (b), PVA/XG-80/20-2 (c), PVA/XG-80/20-3 (d).

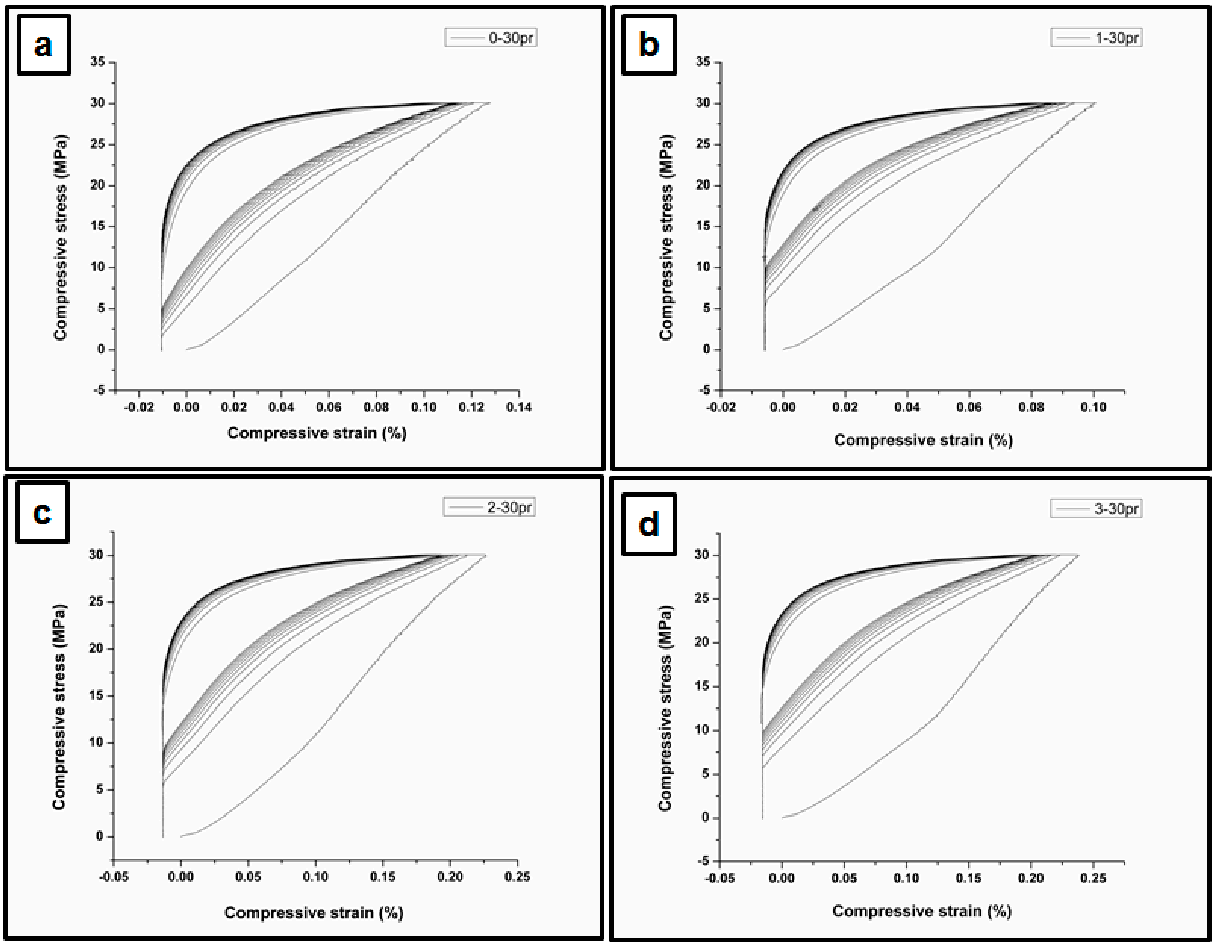

Figure S3. Compressive stress–strain curves of the studied PVA/XG-80/20 hydrogels in the series under 30% compression at room temperature; PVA/XG-80/20 (a), PVA/XG-80/20-1 (b), PVA/XG-80/20-2 (c), PVA/XG-80/20-3 (d).

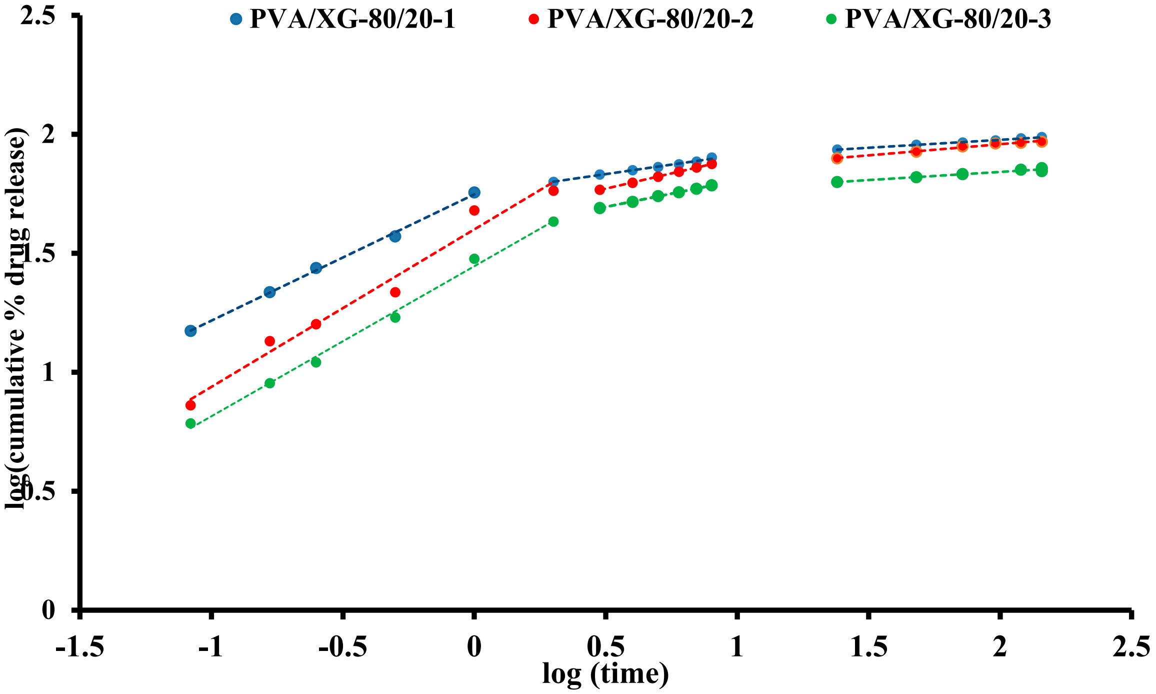

a) Korsmayer-Peppas

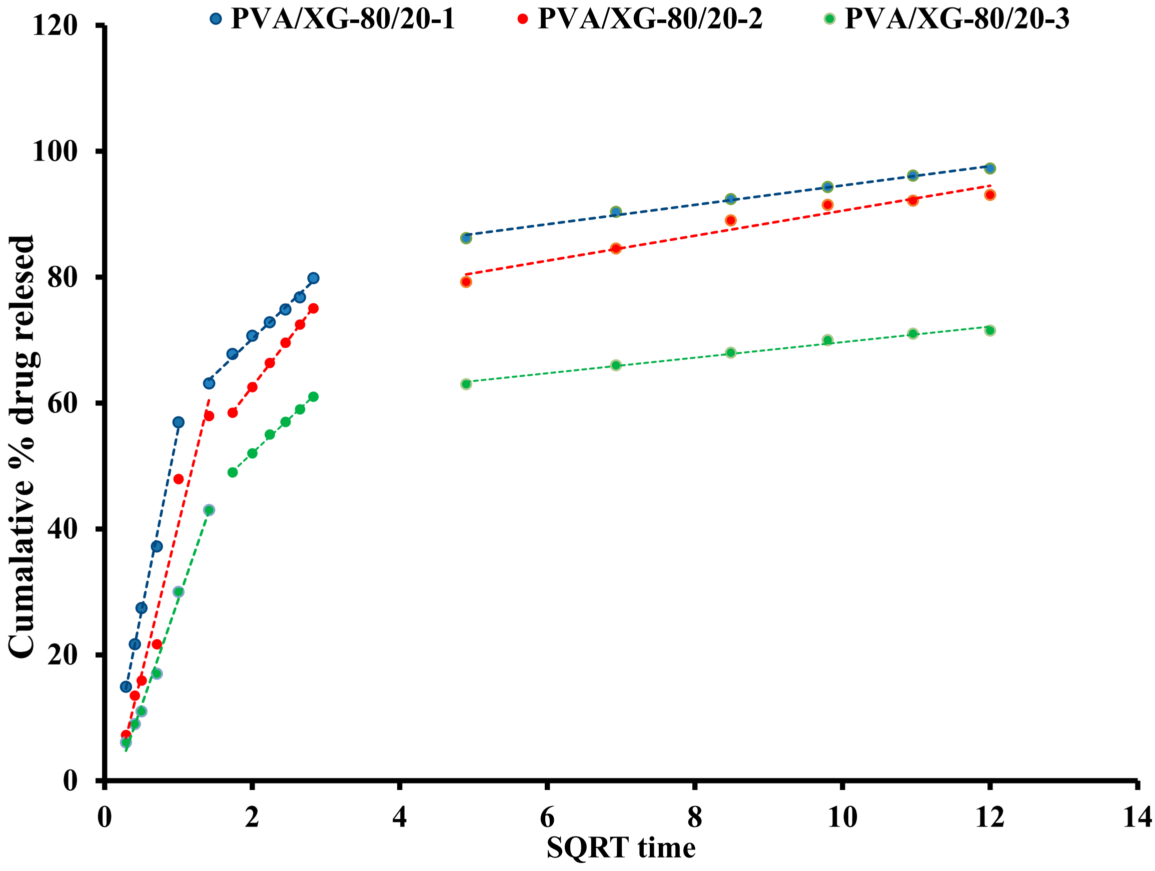
PVA/XG-80/20-1
PVA/XG-80/20-2
PVA/XG-80/20-3
Cumalative % drug relesed
SQRT time
0
20
40
60
80
100
120
0
2
4
6
8
10
12
14

b) Higuchi

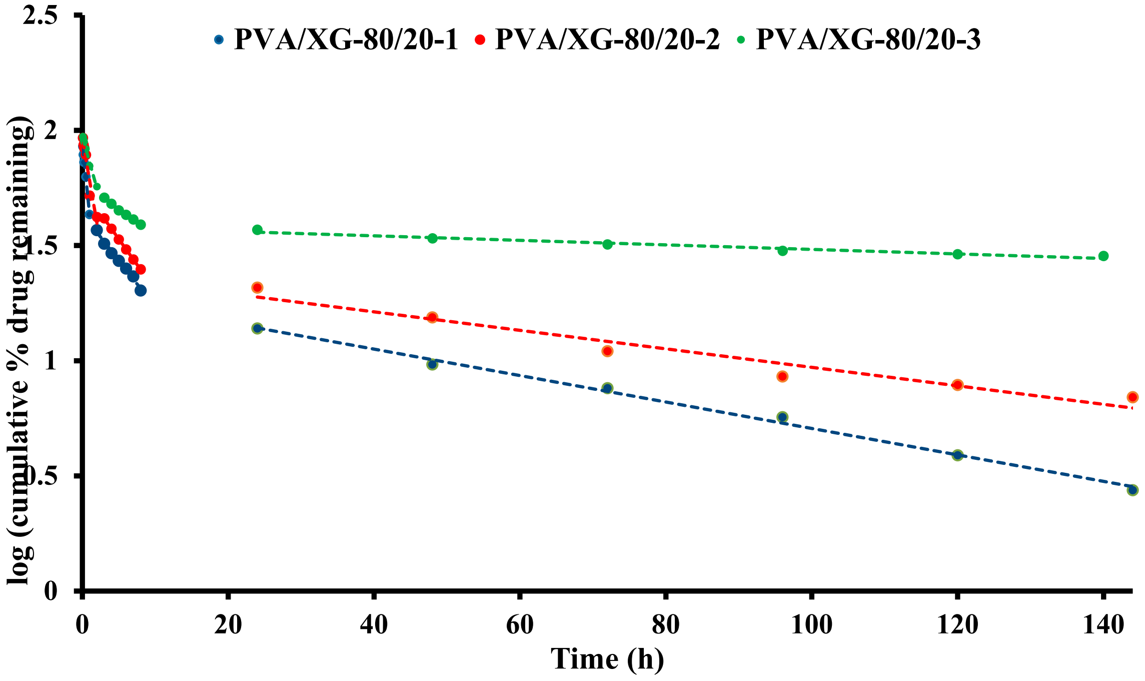

c) First Order

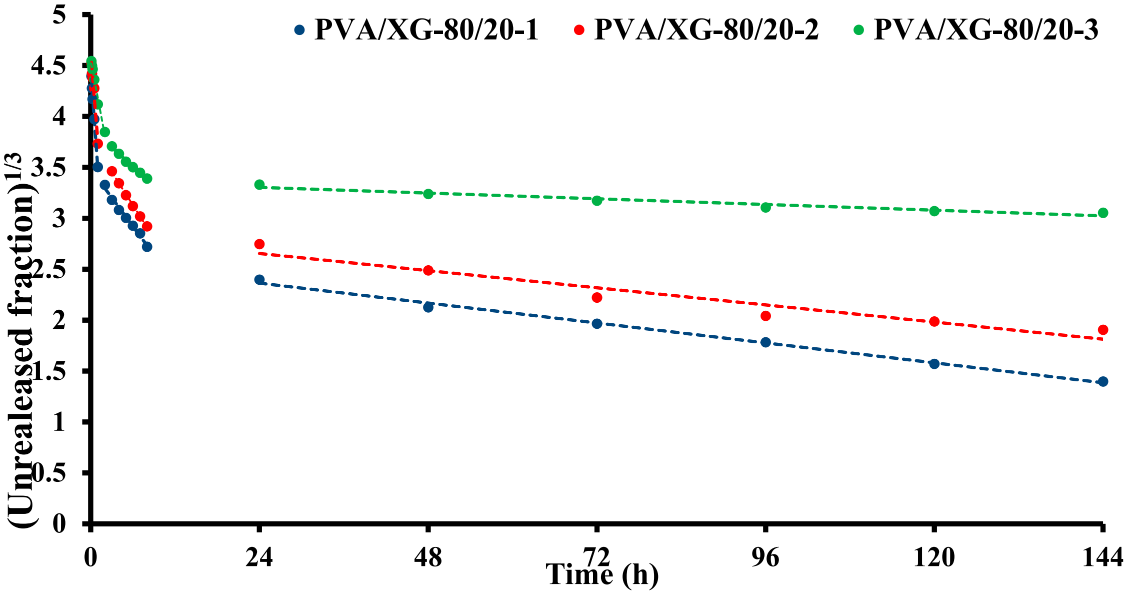

d) Hickson-Crowel

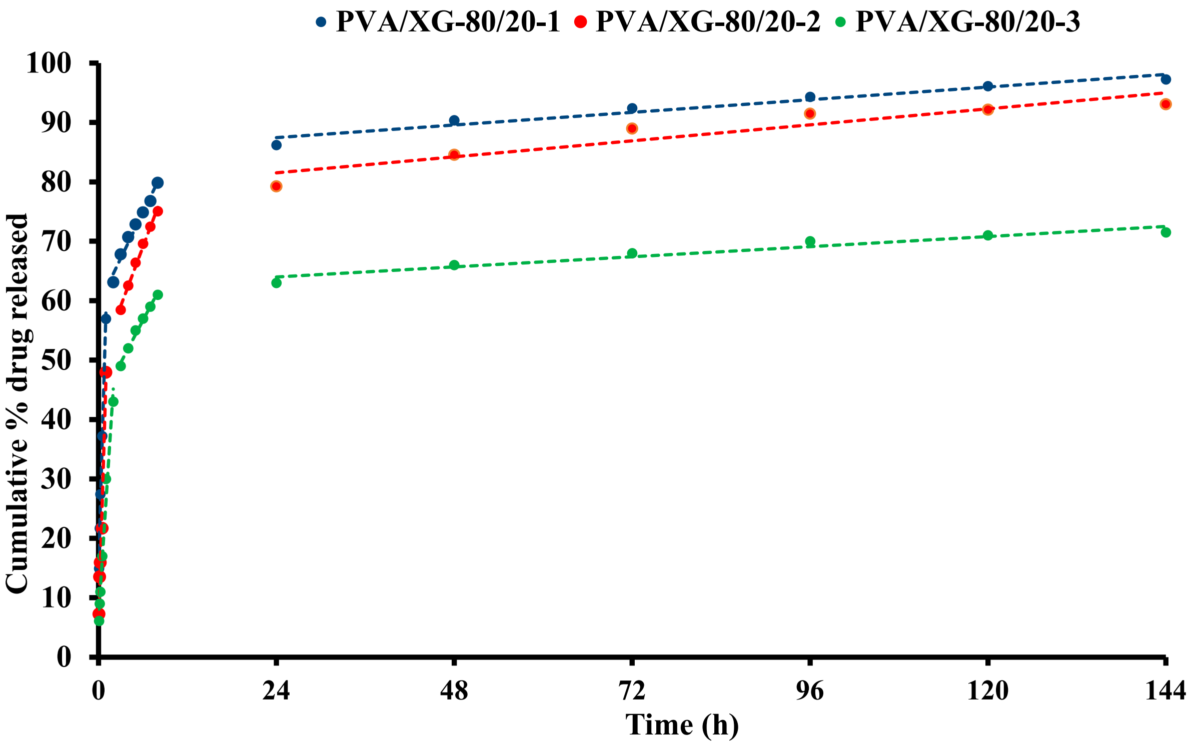

e) Zero Order

Figure S4. Different mathematical models fitted on the *in vitro* release profile on each of the three stages

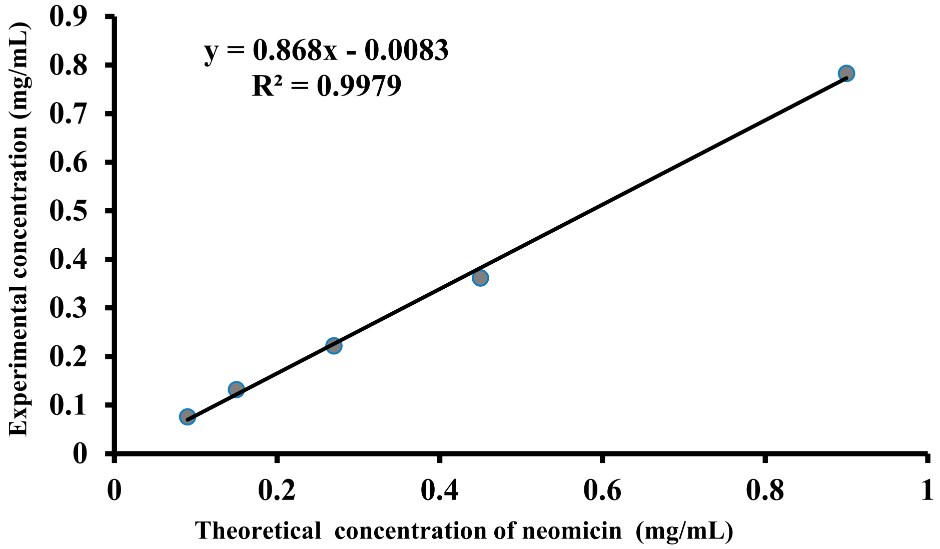

Figure S5. Calibration curve as obtained from NMR data
